# Cholinergic Hypofunction in Presbycusis-Related Tinnitus With Cognitive Function Impairment: Emerging Hypotheses

**DOI:** 10.3389/fnagi.2018.00098

**Published:** 2018-04-06

**Authors:** Qingwei Ruan, Zhuowei Yu, Weibin Zhang, Jian Ruan, Chunhui Liu, Ruxin Zhang

**Affiliations:** ^1^Shanghai Institute of Geriatrics and Gerontology, Shanghai Key Laboratory of Clinical Geriatrics, Huadong Hospital, and Research Center of Aging and Medicine, Shanghai Medical College, Fudan University, Shanghai, China; ^2^Tongji Medical College, Huazhong University of Science and Technology, Wuhan, China; ^3^Department of Otolaryngology, Huadong Hospital, Shanghai Medical College, Fudan University, Shanghai, China

**Keywords:** presbycusis, tinnitus, cognitive impairment, cholinergic hypofunction, glial cell, neurogliaform cell

## Abstract

Presbycusis (age-related hearing loss) is a potential risk factor for tinnitus and cognitive deterioration, which result in poor life quality. Presbycusis-related tinnitus with cognitive impairment is a common phenotype in the elderly population. In these individuals, the central auditory system shows similar pathophysiological alterations as those observed in Alzheimer’s disease (AD), including cholinergic hypofunction, epileptiform-like network synchronization, chronic inflammation, and reduced GABAergic inhibition and neural plasticity. Observations from experimental rodent models indicate that recovery of cholinergic function can improve memory and other cognitive functions via acetylcholine-mediated GABAergic inhibition enhancement, nicotinic acetylcholine receptor (nAChR)-mediated anti-inflammation, glial activation inhibition and neurovascular protection. The loss of cholinergic innervation of various brain structures may provide a common link between tinnitus seen in presbycusis-related tinnitus and age-related cognitive impairment. We hypothesize a key component of the condition is the withdrawal of cholinergic input to a subtype of GABAergic inhibitory interneuron, neuropeptide Y (NPY) neurogliaform cells. Cholinergic denervation might not only cause the degeneration of NPY neurogliaform cells, but may also result in decreased AChR activation in GABAergic inhibitory interneurons. This, in turn, would lead to reduced GABA release and inhibitory regulation of neural networks. Reduced nAChR-mediated anti-inflammation due to the loss of nicotinic innervation might lead to the transformation of glial cells and release of inflammatory mediators, lowering the buffering of extracellular potassium and glutamate metabolism. Further research will provide evidence for the recovery of cholinergic function with the use of cholinergic input enhancement alone or in combination with other rehabilitative interventions to reestablish inhibitory regulation mechanisms of involved neural networks for presbycusis-related tinnitus with cognitive impairment.

## Introduction

Subjective tinnitus, mainly induced by hearing loss and emotional states, is heterogeneous, affecting the development of effective intervention strategies. Presbycusis, commonly referred to as age-related hearing impairment, is a potential risk factor for tinnitus (Shargorodsky et al., 2010; Knipper et al., 2013) and cognitive impairment, including Alzheimer’s disease (AD) and non-AD dementia (Lin et al., 2011, 2013; Bakhos et al., 2015; Panza et al., 2015a,b; Taljaard et al., 2016; Thomson et al., 2017). Thus, presbycusis-related tinnitus and cognitive impairment often appear stimulaneously within a subset of the elderly population.

Epidemiological studies have shown that the prevalence of both presbycusis and dementia increases with age. Approximately one-third of individuals over 65 years of age experience hearing loss greater than 40 dB (averaged across 0.5–4 kHz), more than 10% experience dementia, and more than 90% of individuals with dementia have hearing abnormalities (Marti et al., 2014). Presbycusis is associated with cognitive decline and late-life cognitive disorders due to peripheral hearing impairment (Gates and Mills, 2005; Wallhagen et al., 2008; Gallacher et al., 2012; Lin et al., 2013; Behrman et al., 2014; Deal et al., 2017; Loughrey et al., 2018) or central auditory processing dysfunction (Gennis et al., 1991; Gates et al., 2002, 2011). A prospective epidemiological cohort study showed that observed hearing loss was associated with a greater risk of incident dementia in a multiethnic population (*n* = 1881) followed up over a mean of 7.3 ± 4.4 years (Golub et al., 2017). Moreover, case-control and population-based studies have shown that patients with mild cognitive impairment (MCI), dementia, and AD also have central auditory processing dysfunction and topographically specific neurodegeneration resulting from amyloid senile plaques (SP) and neurofibrillary tangles (NFTs; Sinha et al., 1993; reviewed by Panza et al., 2015a,b).

It is difficult to establish a causal relationship between presbycusis and age-related cognitive decline. Nonetheless, hearing loss could be an early symptom of cognitive decline in elderly individuals, and therefore an appropriate component of screening tools for preclinical diagnosis (Wong et al., 2014). Presbycusis also could be seen as a modifiable factor for preventing cognitive impairment (Lin, 2011; Lin et al., 2011; Gurgel et al., 2014; Marti et al., 2014; Panza et al., 2015a,b). Indeed, timely hearing rehabilitation at the preclinical stage of cognitive decline, including hearing aids and/or cochlear implants, may act to suppress tinnitus and protect cognition by reducing social isolation and depression, reversing maladaptive neuronal plasticity, and improving neurotrophic support and working memory (Acar et al., 2011; Langguth et al., 2013; Marti et al., 2014; Panza et al., 2015a,b; Shore et al., 2016). A whole body of literature indicates that there is no causal relationship between hearing loss and general cognitive loss. Presentation of two age-related disorders together could purely reflect the fact that both conditions are more common in elderly individuals.

Epidemiological studies have also reported that the prevalence of tinnitus increases with age and is highest in elderly individuals aged 60 and 69 years (Adams et al., 1999; Ahmad and Seidman, 2004). The most common symptom of tinnitus is cognitive deficits (Andersson et al., 1999; Hallam et al., 2004; Andersson and McKenna, 2006; Pierce et al., 2012), including working memory and processing speeds on neurocognitive testing (Rossiter et al., 2006), cognitive efficiency (Hallam et al., 2004) and attention control (Stevens et al., 2007). The prevalence of cognitive deficits in patients with tinnitus is higher than would be expected by chance. Approximately 70% of patients with tinnitus had self-reported difficulty concentrating (Andersson et al., 1999). Compared with healthy controls and those with acquired hearing loss, patients with tinnitus also report a greater number of cognitive impairments (Hallam et al., 2004). However, individuals with normal-hearing and tinnitus report similar cognitive performance with individuals with normal hearing without tinnitus (Waechter and Brännström, 2015).

Presbycusis-related tinnitus and cognitive impairment are associated with aging. The former may reflect an independent pathological process that shares some etiologies and pathophysiological alterations with cognitive decline (Marti et al., 2014). The ApoE ε4 allele is a genetic risk factor for both age-related hearing loss (Kurniawan et al., 2012) and AD (Hollands et al., 2017). Cholinergic hypofunction, chronic inflammation and vascular factors are probably linked to the pathogenesis of both presbycusis-related tinnitus and age-related cognitive impairment (Benzing et al., 1993; Emre et al., 1993; Shulman et al., 2008; Daulatzai, 2010; Haase et al., 2011; Fortunato et al., 2016; Wu and Chiu, 2016; Panza et al., 2017). Particularly, cholinergic hypofunction related to aging can aggravate functional deficits of GABAergic interneurons, NFTs, chronic systemic inflammation, age-related blood-brain barrier dysfunction and maladaptive plasticity resulting in an increased spontaneous firing rate, synchronized epileptic-like neuronal activity and excitotoxicity (Knipper et al., 2013; Shore et al., 2016).

While the majority of studies that we refer to are based on animal models, age-related degeneration of synapses and neural anatomy in the peripheral and central nervous system (CNS) may represent a common neurophysiological basis of presbycusis-related tinnitus and age-related cognitive impairment. We hypothesize that age-related loss of cholinergic innervation of various brain structures may be a common link between tinnitus seen in presbycusis-related tinnitus and age-related cognitive impairment. Recovery of cholinergic function may be useful to treat presbycusis-related tinnitus with cognitive impairment by affecting multiple shared pathophysiological targets.

### Declining Cholinergic Function in Humans With Presbycusis-Related Tinnitus and Age-Related Cognitive Impairment

Aging and neurodegenerative diseases are the major causes of declining cholinergic function. Aging leads to cholinergic hypofunction of the basal forebrain cholinergic complex, which is the main cholinergic projection to the cerebral cortex and hippocampus. Gradual age-related loss of cholinergic function results from decreased trophic support from nerve growth factor (NGF) and degeneration of dendritic, axonal and synaptic structures, which cause brain function decline, including cognitive impairment (Daulatzai, 2010; Schliebs and Arendt, 2011).

As in normal aging, patients with MCI and early-stage AD only exhibit declining cholinergic function without cholinergic neurodegeneration. Such changes include an imbalance in the expression of NGF, pro-NGF, the high NGF receptor, trkA and low NGF neurotrophin p75 receptor, as well as changes in acetylcholine release and choline uptake (Cohen et al., 1995; Schliebs and Arendt, 2011). The advanced stages of early-onset and late-onset AD and psychiatric disorders (e.g., Parkinson’s disease and Lewy body dementia) are characterized by a severe loss of NGF receptor positive cholinergic cells in the basal forebrain (Mufson and Kordower, 1989; Perry, 1990). NGF receptors play a role in cholinergic neuron death. Decreased expression of NGF receptors was also observed on among striatal cholinergic neurons in the AD brain (Boissière et al., 1996). Furthermore, encapsulated cell implants releasing NGF bilaterally to the basal forebrain of patients with AD across 12 months significantly enhanced cerebrospinal fluid levels of the cholinergic biomarker choline acetyltransferase (ChAT; Karami et al., 2015). Age-related loss of the calcium-binding protein, calbindin-D28K, in basal forebrain cholinergic neurons has been related to the full range of tau pathology of AD (Ahmadian et al., 2015).

Cholinergic hypofunction also involves changes in the presynaptic synthetic enzyme, ChAT and acetylcholine receptor (AChR) expression. In patients with AD compared with age-matched healthy controls, there is a 50%–90% decline in activity of presynaptic ChAT (Perry et al., 1978; Davies, 1979). Moreover, significant declines in enzyme activity that result in cholinergic dysfunction do not occur until a relatively late stage (Davies et al., 1999; Tiraboschi et al., 2000). In contrast, loss of ChAT activity in patients with Lewy bodies was present in the earliest stage (Tiraboschi et al., 2002). In the frontal cortex of individuals with AD, different alterations have been observed in muscarinic (M) subtypes, with diminished M1 and M2 but increased M4 immunoreactivity, and normal M1, decreased M2 and increased M4 numbers of binding sites (Flynn et al., 1995). Cholinergic deficits are associated with the loss or derangement of nicotinic acetylcholine receptors (nAChRs) in the brains of those with AD and Down syndrome (Engidawork et al., 2001), with significantly decreased alpha 7 and significantly increased alpha 3 receptors in the frontal cortex in AD. Autopsy brain tissue (Guan et al., 2000; Lee et al., 2000) and *in vivo* evaluations (Nordberg et al., 1997) of patients with AD have consistently shown decreased nAChR levels. Moreover, after blockade of muscarinic receptors with scopolamine, young healthy individuals have a similar pattern of memory and cognitive decline as aged individuals with cholinergic dysfunction (Drachman et al., 1980). Nicotinic cholinergic blockade with mecamylamine in elderly healthy individuals resulted in AD-like cognitive deficits and specific blood flow abnormalities in the parieto-temporal cortex (Gitelman and Prohovnik, 1992). Therefore, tacrine and nicotine, which stimulate the cholinergic system, could significantly improve attentional function associated with basal forebrain cholinergic innervation of the cortex and other brain regions in patients with AD (Lawrence and Sahakian, 1995).

Degeneration of the basal forebrain cholinergic system due to aging and AD causes impairment of thalamo-cortical function, reduced connectivity between the thalamo-cortical system, hippocampus, and other key brain regions, and decreased cerebral blood flow (CBF), which has been associated with cognitive disturbances and age-related sensory loss (Daulatzai, 2010). The amygdala is a component of the limbic system involved in emotion, attention and memory. Differences have also been observed between the aging human brain and AD in the loss of cholinergic innervation of the amygdaloid complex (Benzing et al., 1993; Emre et al., 1993). Compared with middle-aged controls, no decline in cholinergic input of the amygdale was observed in immunohistological specimens from aging participants (Emre et al., 1993). Another study reported that individuals without dementia but with high rates of SP showed highly dystrophic neurites, but no significant loss of fiber innervations (Benzing et al., 1993). However, there does appear to be a severe and regionally selective loss of cholinergic innervations in the amygdaloid complex of patients with AD.

Cholinergic hypofunction results in impairments of the auditory pathway, as well as impaired cortico-cortical interactions between auditory and other sensory regions. In patients with mild to moderate AD, dysfunction is observed in the primary auditory pathway and ascending reticular activating system, which have cortical cholinergic innervation. Furthermore, significant delays in I~V interpeak latency of brain auditory evoked responses and dysfunction in the generation of primary auditory cortex evoked potentials, as well as reduced neuronal activity in the ascending reticular activating system are observed in AD (O’Mahony et al., 1994). There is a progressive decline in the attenuation of subsequent auditory evoked potentials by a visual stimulus from the young to the healthy elderly to individuals with MCI and AD (Golob et al., 2015). However, in the human cochlear nucleus, nAchR beta 2 immunostaining was unchanged from birth to 90 years (Sharma et al., 2014). Based on observations from human studies, the loss of cholinergic innervation to various brain structures may provide a link between tinnitus seen in presbycusis-related tinnitus and age-related cognitive impairment. Recovery of cholinergic function during an optimal time window before the loss of cholinergic neurons may therefore lead to better outcomes.

### Declining Cholinergic Function May Contribute to the Accumulation of Beta-Amyloid Oligomers and NFTs in Age-Related Cognitive and Hearing Impairments

The neuropathological hallmarks of AD, including amyloid deposits and tau-immunoreactive NFTs, are also present in the healthy aging brain. An immunohistological study of serial sections from 105 autopsy brains of cognitively normal patients (age range: 40–104 years) showed that NFTs appear earlier than amyloid plaques during normal aging. All cases from people over 48 years old displayed at least a few NFTs (more frequently in the entorhinal than in the transentorhinal cortex), which was preceded by tau pathology in these areas rather than in the brainstem (Tsartsalis et al., 2018). In the auditory system of individuals with AD, the ventral nucleus of the medial geniculate body and central nucleus of the inferior colliculus show SP and NFT distributions with a topographically specific and consistent pattern of degeneration (Sinha et al., 1993). Significant age-related reductions in calcium binding proteins has been observed in later decades in the ventral cochlear nucleus, which is similar to results for cholinergic neurons of the basal forebrain in patients with AD, and might be related to tau pathology (Sharma et al., 2014; Ahmadian et al., 2015).

Noise exposure is a common cause of tinnitus and hearing impairment. Animal research shows that exposure to moderate intensity white noise (80 dB SPL, 2 h/day) can impair learning and memory in mice (Cheng et al., 2011). Moreover, it has been demonstrated that the hippocampus is more susceptible to noise than is the auditory cortex (Cheng et al., 2016). Indeed, significant increases in peroxidation and tau hyperphosphorylation in the hippocampus have been observed after a week of noise exposure, but there were no increases in the auditory cortex 3 weeks after exposure. Chronic white noise (100 dB SPL, 4 h/day × 14 day) persistently increased tau hyperphosphorylation at the same sites that are typically phosphorylated in the AD brain and glycogen synthase kinase 3β (GSK3β), as well as increased the formation of pathological NFT tau in the hippocampus and prefrontal cortex (Cui et al., 2012). Such changes in the frontal cortex also play an important role in the pathogenesis of frontal dementia, while changes in the frontal acoustic cortex are seen in the early onset of communication deficiency (Baloyannis et al., 2001).

Tau hyperphosphorylation sequesters normal tau and microtubule-associated proteins into insoluble NFTs and inhibits microtubule assembly (Iqbal et al., 2013). Tau reduction prevents cognitive decline, synaptic transmission and plasticity, and spontaneous epileptiform activity in AD model mice that overexpress Aβ, without changing the expression of Aβ (Ittner et al., 2010). Furthermore, tau-deficient AD models have demonstrated a reversal in the Aβ induced imbalance of excitation/inhibition, NMDA receptor dysfunction, and excitotoxicity in both transgenic and wild type mice (Roberson et al., 2007, 2011).

Loss of cholinergic innervations may play important roles in both AD and hearing impairment during aging. The AChE inhibitor donepezil can protect against Aβ induced neurotoxicity by enhancing protein phosphatase 2A (PP2A) activity and inhibiting GSK3β activity via the activation of nAChRs, which reduces tau-induced neuronal toxicity and neurodegeneration (Bitner et al., 2009; Noh et al., 2009). In the brain, mAChRs may mediate cognitive function and neuropsychiatric symptoms and they are also considered potential targets in AD and schizophrenia (Clader and Wang, 2005; Poulin et al., 2010; Foster et al., 2014). M1 type mAChRs, mainly present in the striatum, hippocampus and neocortex, are activated by M1 specific agonists doses without adverse effects. Such activation could improve learning, memory, synaptic plasticity, and cognitive functions via the activation of extracellular signal-regulated kinases (Berkeley et al., 2001; Ragozzino et al., 2012). In A7KO-APP AD transgenic mice, the absence of alpha-7 nAChRs leads to Aβ accumulation and oligomerization, exacerbating early-stage cognitive decline and septohippocampal pathology (Hernandez et al., 2010).

### Cholinergic Denervation of NPY Neurogliaform Cells May Be Involved in Presbycusis-Related Tinnitus With Cognitive Impairment

Reduced functional connectivity in the brains of patients with AD or MCI, as well as the elderly with cognitive complaints or cognitively normal ApoEε4 carriers, reflects activity changes within the default-mode network, which is most active at rest and deactivated during cognitive tasks (Ruan et al., 2016). The loss of cholinergic innervations and reduced GABAergic inhibition might play important roles in such changes.

Distinct GABAergic cell types project to the surface of pyramidal cells in the cortex and hippocampus, forming neural circuits for inhibitory control of brain function and plasticity. Functional remodeling of GABAergic neurotransmission has been observed in the human brain with AD (Limon et al., 2012). Moreover, GABA currents in the temporal cortex of the AD brain show age-related reductions, which were associated with reduced mRNA and protein for the main GABA receptor subunits. In the ADbrain compared with controls, α1 and γ2 transcription shows down-regulation, while but α2, β1 and γ1 transcription shows up-regulation. In patients with AD and/or epilepsy, deficits of GABAergic interneurons are associated with aberrant network activity, including hyperexcitability, clusters of hyperactive and hypoactive neurons, and network/spontaneous epileptiform activity (Olney, 1995; Nägerl et al., 2000; Snider et al., 2005; Palop and Mucke, 2009).

Patients with tinnitus show alterations in global brain networks, including decreased default-mode network activity, and increased activation of the auditory cortex and amygdala (Schlee et al., 2009; Elgoyhen et al., 2015). These alterations may result from decreased fuctional connectivity from peripheral and other brain regions. Tinnitus may be a consequence of maladaptive plasticity-induced disturbances of excitation-inhibition homeostasis with net down-regulation of inhibitory neurotransmission in the central auditory pathway. Subsequently, the central auditory system compensates for decreased input by up-regulating network activity among central circuits (Salvi et al., 2000; Knipper et al., 2013; Shore et al., 2016). Decreased peripheral input induced by auditory trauma and aging leads to altered cortical activity patterns, including increased spontaneous firing rates, synchronized epileptic-like neuronal activity, and basal excitatory postsynaptic potentials (for a review, see Knipper et al., 2013). Plastic tinnitus-related changes include loss of glycinergic inhibition in the adult dorsal cochlear nucleus and/or loss of GABAergic inhibition in the inferior colliculus and higher centers, resulting in aberrant cortical activity patterns (Wang et al., 2011).

Although cholinergic drugs can temporarily suppress tinnitus in some patients, these interventions cannot eliminate the pathological neural activity. Mounting evidence from clinical trials suggests that vagus nerve stimulation (VNS)-based targeted plasticity therapies are effective in patients with neurological diseases (Hays, 2016). VNS in combination with auditory stimulation can reverse pathological neuroplastic changes of the auditory cortex toward physiological neural activity and synchronicity via M cholinergic neuromodulation (Engineer et al., 2013; Bojić et al., 2017; Tyler et al., 2017). Based on these studies in humans, GABAergic interneuron deficits in the auditory cortex and limbic system may play a key role in presbycusis-related tinnitus with cognitive impairment.

#### Loss of Cholinergic Innervation and Reduced Inhibition of NPY Neurogliaform Cells in Age-Related Cognitive Impairment

Animals studies have shown that GABAergic interneuron deficits result in aberrant excitatory neuronal activity in mouse AD models (Palop et al., 2007; Roberson et al., 2007, 2011; Verret et al., 2012; Iaccarino et al., 2016). Both nAChRs (Buhler and Dunwiddie, 2002; Maloku et al., 2011; Zappettini et al., 2011) and mAChRs (Pitler and Alger, 1992; Zhong et al., 2003; González et al., 2011; Yi et al., 2014) are expressed in GABAergic interneurons and mediate GABA release from these neurons. Neuropeptide Y (NPY)-neurogliaform (Faust et al., 2015), somatostatin (Faust et al., 2015; Muñoz et al., 2017) and parvalbumin (Yi et al., 2014) subtype interneurons express AChRs and receive cholinergic excitatory input. NPY-neurogliaform cells primarily reside within both the stratum radiatum and lacunosum-moleculare of the hippocampus, as well as the superficial and deep layers of the neocortex, which are significantly decreased in the hippocampus of animal models with AD or seizures (Mazarati and Wasterlain, 2002; Faust et al., 2015). However, optogenetic stimulation of cholinergic fibers in transgenic mice expressing the human ApoE ε4 allele has been shown to abolish partial neuronal loss in the entorhinal cortex induced by abnormal hyperactivity in dentate networks (Bott et al., 2016).

The activation of both the α(7) nAChR and α4β2 nAChR subtypes could enhance GABA release in hippocampal synaptosomes (Zappettini et al., 2011). Furthermore, α(4)β(2) nAChR agonists may control epigenetic alterations induced by glutamic acid decarboxylase 67 (GAD 67) increases in GABAergic neurons better in schizophrenia than do α(7) nAChR agonists (Maloku et al., 2011). M1 mAChRs in parvalbumin interneurons could improve GABAergic transmission in hippocampal and prefrontal cortical pyramidal neurons (Yi et al., 2014). Moreover, activation of M1–M5 mAChRs in rat hippocampal neurons *in vitro* increases GABAergic inhibitory transmission (González et al., 2011). Treatment with Huperzine A leads to robust and sustained seizure resistance in genetic epilepsy models with voltage-gated sodium channel mutation via the activation of mAChRs and GABA_A_ receptors (Wong et al., 2014). However, nAChR-mediated GABAergic cortical inhibition in rats, related to increased high gamma frequency visible on electroencephalogram, might also be involved in the Huperzine A anticonvulsant mechanisms (Gersner et al., 2015). Thus, solely based on the animal models, the loss of cholinergic innervation of NPY-neurogliaform cells in various brain structures contributes to aberrant excitatory neuronal activity in age-related cognitive impairment.

#### Cholinergic Denervation of NPY Neurogliaform Cells in the Central Auditory System in Presbycusis With Tinnitus

In animal studies, changes in inhibitory properties that are induced by aging and acoustic trauma, similar to deafferentation plasticity changes in other mammalian sensory systems, have been observed from the cochlear nuclei to the auditory system. The cochlear nuclei of aged rats have lower glycine levels and altered glycine receptor subunit compositions compared with young rats (Banay-Schwartz et al., 1989). However, in the inferior colliculus of rats, age-related loss of GABAergic inhibition caused by the loss of the biosynthetic enzyme GAD, as well as reduced GABA levels and GABA release, may be involved in the abnormal perception of signals in noise and the deterioration of speech discrimination (Milbrandt et al., 1994, 2000; Raza et al., 1994).

Age-related decreases in GAD have been observed in the primary auditory cortex, parietal cortex and hippocampus, with more significant reductions observed in the auditory cortex of rats (Stanley and Shetty, 2004; Ling et al., 2005). Age-related alterations in GABA receptor subunit composition have also been observed in the inferior colliculus and primary auditory cortex of aged rats, such that there are changes to the wild-type receptor proportions (Caspary et al., 2013). These presynaptic and postsynaptic changes may contribute to increased spontaneous activity in neurons of the inferior colliculus and layer-specific increases in the spontaneous activity of the primary auditory cortex (Ling et al., 2005). Following sound exposure in rats with tinnitus, single units within the medial geniculate body of rats exhibited enhanced spontaneous firing, altered burst properties, and increased rate-level function slopes, which acts to alter sensory gating and enhance the gain of neuronal networks in the auditory cortex and limbic centers (Kalappa et al., 2014).

**Figure 1:**
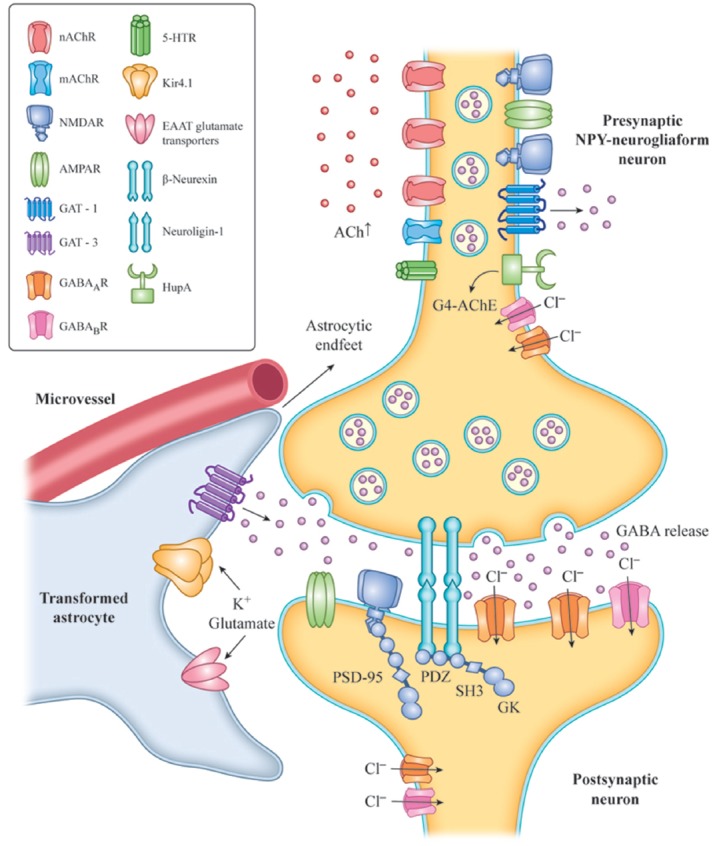
Increased level of Ach with HupA inhibition of AChE activation or VNS, resulting in activation of AChRs, in NPY-neurogliaform neurons.The increased GABAergic signaling may regulate inhibitory tone and network activity by phasic or synaptic transmission, tonic activation and volume transmission. The adhesion complex of Neuroligin-1 (NL1) and β-neurexin is involved in the maintenance of synapses. The C-terminal fragment of NL-1 and NMDA receptors interacts with the PDZ domains of PSD-95 in the postsynaptic region of neurons, and mediates excitatory synaptic efficacy and plasticity. Transformed astrocytes contribute to neuron hypersynchronicity and excitability, which mainly occurs by reduced expression of potassium inward-rectifying channels (Kir4.1), reduced gap junctions, impaired glutamate metabolism and increased release of inflammatory mediators. VNS, vagus nerve stimulation; NPY, neuropeptide Y; G4-ACh, tetrameric acetylcholinesterase; AChR, acetylcholine receptor; EAAT, glutamate transporter of astrocytes; and GAT, GABA transporter.

Inhibitory transmission and survival of NPY-neurogliaform cells in the hippocampus and prefrontal cortex is mainly under cholinergic regulation in experimental rodents (Mazarati and Wasterlain, 2002; Faust et al., 2015; Overstreet-Wadiche and McBain, 2015; Bott et al., 2016). Therefore, we hypothesized that withdrawal of nicotinic cholinergic input to NPY neurogliaform cells is a key component of the pathological mechanism underlying presbycusis with tinnitus and cognitive impairment, solely based on animal models. The enhancement of GABA release from NPY-neurogliaform cells and the reversal of the imbalance between excitation and inhibition in the central auditory system following the recovery of cholinergic function may provide an important target for interventions to treat presbycusis with tinnitus (Figure 1).

Beyond the central auditory system, axosomatic synapses between the medial olivocochlear efferent system and outer hair cells are cholinergic. A feedback system eliciting efferent suppression via alpha-9/alpha-10 nAChRs can improve the detection of signals in background noise, enable selective attention to particular signals, and protect the periphery from damage caused by overly loud sounds (Maison et al., 2002; Elgoyhen et al., 2009). Our previous animal studies have shown that aging and ototoxic drugs exacerbate the degeneration of the mouse medial olivocochlear efferent system (Ruan et al., 2014a,b,c). Furthermore, histopathological studies of the human cochlear have shown that those with presbycusis and tinnitus had a significantly greater loss of outer hair cells in the basal and upper middle turns, and greater atrophy of the stria vascularis in the basal turn compared with those with presbycusis without tinnitus (Terao et al., 2011). Therefore, nAChR activation in the peripheral medial olivocochlear efferent system may also play a role in the suppression of presbycusis with tinnitus.

### Nicotinic Denervation Induced Immuno-Dysregulation May Involved in Presbycusis-Related Tinnitus With Cognitive Impairment

Observations from clinical studies indicate that, glial cell activation and chronic systemic inflammation during normal and pathologic brain aging are related to poor cognitive performance and a risk of cognitive decline in dementia, vascular dementia, and AD (Schmidt et al., 2002; Weaver et al., 2002; Engelhart et al., 2004; Yaffe et al., 2004). Inflammation plays a critical role in the fluctuation of non-cognitive neuropsychiatric symptoms (Kat et al., 2008; van Gool et al., 2010). Indeed, free radical-induced oxidative damage and chronic inflammation play important roles in the development of dysfunctional connections between the central cortex and the inner ear in hearing disorders (Haase et al., 2011).

Age-related increase in GFAP positive glial cells have been observed in the cochlear nucleus (Sharma et al., 2014). In a cross-sectional cohort of 360 community-dwelling individuals aged 60 years and over, increased inflammatory markers and white blood cell count were associated with worsening presbycusis, with the strongest positive correlation seen in those over 75 years (Verschuur et al., 2014). Furthermore, the inflammatory cytokine TNF-α (rs1800630) and the TNF receptor superfamily 1B (rs1061624) have been related to an increased risk of hearing damage in a population-based cohort study of elderly Japanese individuals (Uchida et al., 2014).

Chronic inflammation also leads to blood brain barrier (BBB) vulnerability and brain hypoperfusion. Increased release of neurotoxic and inflammatory mediators has been observed in the brain microvessels of patients with AD (Grammas, 2011). Further, chronic inflammation causes BBB dysfunction and increased vascular permeability during aging, as well as in AD and other neurodegenerative disorders (Farrall and Wardlaw, 2009; Erdő et al., 2017). Moreover, the loss of cholinergic innervation to the basal forebrain results in decreased CBF (Martin et al., 1991; Daulatzai, 2010). Compared with neurologically healthy individuals without the ApoE ε4 allele, those with the ApoE ε4 allele show greater regional CBF reductions in the brain, making it vulnerable to pathological alterations in AD (Thambisetty et al., 2010; Hollands et al., 2017) and presbycusis (Kurniawan et al., 2012).

These results suggest that chronic inflammation and hypoperfusion play important roles in the pathogenesis of presbucusis-related tinnitus with cognitive impairment. Recovery of cholinergic function with AChE inhibitors, including donepezil, tacrine, pyridostigmine, galantamine, rivastigmine and Huperzine A shows potential disease-modifying benefits in the treatment of neuropsychiatric symptoms in patients with AD (Linton, 2005; Rafii et al., 2011) and dementia (Freund-Levi et al., 2014), as well as for the musical hallucinations that occur with hearing loss (Ukai et al., 2007; Zilles et al., 2012; Blom et al., 2014, 2015) or hearing loss with tinnitus (Strauss and Gertz, 2009). However, there is no mechanistic explanation for the relationship between cholinergic hypofunction and chronic inflammation alterations in presbucusis-related tinnitus with cognitive impairment.

#### Loss of Cholinergic Innervation and Chronic Systemic Inflammation in Age-Related Cognitive Impairment

Observations from experimental rodent models indicate that anticholinergic activity might initiate and/or accelerate AD pathology in the tauopathy mouse model by enhancing neuroinflammation, including microglial activation. The recovery of lost cholinergic innervation or function by the cholinesterase inhibitor donepezil or Huperzine A could alleviate tau pathology as well as age- and AD-related chronic neuroinflammation (Yoshiyama et al., 2015), and D-galactose-induced neurovascular damage (Ruan et al., 2014d). Moreover, chronic inflammation induced cognitive decline in rats with cerebral hypoperfusion (Wang et al., 2010).

The mechanisms underlying cholinergic anti-inflammation were first observed in human immune cells (Wang et al., 2003). The observations suggested that nicotinic activation of α7nAChR in human macrophages or monocytes is necessary to attenuate the systemic inflammatory response and inhibit the production of proinflammatory mediators by suppression of I-κB phosphorylation and nuclear factor-κB transcriptional activity (Wang et al., 2003; Yoshikawa et al., 2006).

Subsequently, a similar anti-inflammatory mechanism was also observed in rat CNS. Increased brain ACh induced by Huperzine A activates cholinergic-mediated suppression of nuclear translocation of NF-κB, as well as inducing oxidative stress, glial cell activation, and neuroinflammation in rats with ischemia (Wang et al., 2008). Huperzine A combines tetrameric AChE (G4) and indirectly activates both muscarinic and nicotinic types of AChRs (Wang et al., 2010). Moreover, the obvious overlap of tetrameric AChE and α7nAChRs in the hypothalamus, hippocampus, amygdale, cerebral cortex and midbrain of humans and rats (reviewed by Damar et al., 2017) indicates that cholinergic anti-inflammatory effects occur mainly via α7nAChRs in glial and neuronal cells (Pavlov and Tracey, 2006; Wang et al., 2008).

The activation of α7nAChRs in neural cells suppresses central inflammatory responses in mice with Parkinson disease (Stuckenholz et al., 2013), stroke (Han et al., 2014), or traumatic brain injury (Kelso and Oestreich, 2012), and also suppresses glutamate-induced neurotoxicity *in vitro* (Shimohama et al., 1998; Iwamoto et al., 2013). Futhermore, the activation of α7nAChRs in astrocytes down-regulates Aβ1–42-induced increases in NF-κB in *in vitro* (Xie et al., 2016), and improves neurotrophic cytokine S100B secretion, which is decreased in the cerebrospinal fluid in rat models of dementia (Lunardi et al., 2013). Moreover, the upregulation of α7nAChR expression induced by neuregulin in microglial cells suppresses neuroinflammation *in vitro* (Mencel et al., 2013). Based on the above results from clinical and animal studies, loss of cholinergic innervations results in reduced cholinergic anti-inflammatory effects and glial activation, which further aggravates the loss of GABAergic interneurons. Therefore, we hypothesize that the withdrawal of nicotinic cholinergic input induces chronic inflammation, acting as another key step in the pathological mechanism underlying presbycusis with tinnitus and cognitive impairment.

#### Induction of Immuno-Dysregulation by Nicotinic Denervation in the Central Auditory System May Contribute to Presbycusis-Related Tinnitus With Cognitive Impairment

Animal research suggests that auditory cortical cholinergic inputs from the basal forebrain in adult ferrets contribute to cognitive functions related to the processing of auditory stimuli, including normal auditory perception and adaption to changes in spatial cues (Leach et al., 2013). Furthermore, the central auditory pathway, including the inferior colliculus and nuclei of the lateral lemniscus, but not the cochlear nucleus, show significantly reduced ChAT activity in aged Fischer-344 rats (Raza et al., 1994). A significant decrease in muscarinic receptors, but not ChAT activity, in the dorsal hippocampi of aged rats has also been observed (Lippa et al., 1980). Moreover, noise-induced hyperactivity in fusiform cells of the dorsal cochlear nucleus of adult male Syrian golden hamsters has been shown to be inhibited by the cholinergic agonist carbachol (Manzoor et al., 2013). There is also evidence in experimental animals that chronic inflammation contributes to the dysfunction of auditory pathways (Haase et al., 2011; Menardo et al., 2012; Tan et al., 2016). Acute and chronic noise exposure in C57BL/6 mice (Tan et al., 2016) and senescence-accelerated mouse prone 8 mice (Menardo et al., 2012) also results in increased inflammatory responses in the cochlea.

Chronic inflammation leads to BBB dysfunction and increased vascular permeability during aging, as well as in AD and other neurodegenerative disorders (Zlokovic, 2011; Takeda et al., 2014; Erdő et al., 2017). Increased vascular permeability facilitates the spread of peripheral inflammation into the brain and causes more severe non-cognitive symptoms in AD animal models (Takeda et al., 2013), as well as brain hypoperfusion (Zlokovic, 2011; Takeda et al., 2013). A prominent alteration following BBB breakdown is the decrease in the levels of tight junction proteins, which has been observed in an aging animal model and dementia-related diseases (Zlokovic, 2008; Kalaria, 2010; Ruan et al., 2014d).

Loss of cholinergic input during aging and neurodegenerative diseases causes decreased ACh release and brain hypoperfusion. Reduced sensory input can also lead to decreased ACh release in the neocortex and hippocampus (Penschuck et al., 2002), and decreased hippocampal blood flow (Cao et al., 1992). Hypoxia and ischemia clearly contribute to the pathogenesis of sensorineural tinnitus, and some agents can effectively suppress tinnitus by improving the blood supply and inhibiting chronic inflammatory damage in the acute stage (Mazurek et al., 2006). CBF reductions and hypoxia may not only result in the accumulation of hyperphosphorylated tau and filament formation in experimental animals (Gordon-Krajcer et al., 2007), but also cause increased β-secretase transcription (Zhang et al., 2007), decreased Aβ clearance due to loss or oxidization of lipoprotein receptors in endothelial cells and astrocytes (Bell et al., 2009; Owen et al., 2010), Reduced glutamate reuptake by astrocytes (Boycott et al., 2007), and the accumulation of oxidative damage in the vascular endothelium and high metabolic neurons (Fernández-Checa et al., 2010; Figure 1).

Based on animal research, we hypothesize that the cholinergic anti-inflammation mediated by α7nAChR may be one potential mechanism by which hearing loss occurs with tinnitus or cognitive impairment. AChE inhibitors might suppress presbycusis accompanied by tinnitus and may indirectly protect auditory and cognitive function by activating α7nAChR-mediated anti-inflammatory effects in various cells of the brain’s neural vascular unit. This might include the suppression of glial and endothelial activation, neuroinflammation, tau-induced neurotoxicity and decreased gap junctions, as well as improved glutamate and extracellular potassium reuptake by astrocytes. These effects inhibit network hyperexcitability and excitotoxicity in the auditory pathway (Figure 1).

## Conclusion

Presbycusis is a risk factor for tinnitus and cognitive decline. Cholinergic hypofunction might be a major contributor to presbycusis-related tinnitus and age-related cognitive impairment. Cholinergic denervation in the CNS, might lead to the reduction of both inhibition by NPY neurogliaform cells and cholinergic anti-inflammatory effects on the neural vascular unit mediated by nAChRs, as well as suppression of GSK3β activity and tau-induced neurodegeneration.

Implementing VNS and AChE inhibitors alone or in combination with other hearing rehabilitative interventions during the optimal time window may lead to greater disease-modifying benefits in the treatment of presbycusis-related tinnitus with cognitive impairment. However, in the evidence reviewed here, data have mainly been obtained from animal experiments. Age-related hearing loss and AD in humans become apparent very slowly, and are associated with a long preclinical period. Therefore, animal models with a life expectancy of approximately 3 years are not really comparable to humans with these disorders. Further studies are required to elucidate the roles played by M or N cholinergic neuromodulation and distinct GABAergic cell types in the pathophysiological process. Furthermore, it must be investigated whether mechanisms underlying peripheral and central cholinergic regulation are the same.

The potential relationship between tinnitus and depressive systems or affective disorders, and the mechanisms underlying this, should also be investigated in rodents. In addition, dynamic changes in CNS-derived biomarkers of cholinergic hypofunction and neuronal impairment in peripheral body fluids should be investigated as possible screening tools for preclinical or early stage disease, predictors of diagnosis, predictors of intervention outcomes. Finally, innovative, specific and selective neuromodulatory methods and multi-center longitudinal cohort studies are also urgently needed.

## Author Contributions

QR and ZY designed the study and analyzed the data. QR, ZY, WZ, JR, CL and RZ provided a consensus agreement on the final hypotheses and drafted the initial version of the manuscript. WZ, JR and QR collected the data. All authors contributed to the final version of the manuscript.

## Conflict of Interest Statement

The authors declare that the research was conducted in the absence of any commercial or financial relationships that could be construed as a potential conflict of interest.
